# A Home Conversation Made Public or a Housekeeper’s Trouble

**Published:** 1869-04

**Authors:** 


### A Home Conversation made Public

### Or A Housekeeper's Trouble.

"Wife, let us invite Mr. Beecher and wife to tea some night this week."

"Oh, no, don't!" replies wife, "it's *so* much trouble."

"Trouble, nonsense—you need not go to any extra pains to prepare tea for them. Just have a nice clean spread and two more plates than usual, and everything is prepared. Certainly that which is good enough for *us*, aught to be for our company, besides Mr. B. does not approve of those "fancy suppers;" good plain food will be best appreciated by them."

"O, yes, it's easy enough for you men to talk in that manner, but you are always ready to scold if the table does not look as well as other people's. Besides, when folks are asked out to tea, they always look for something nice, and expect that the hostess has been to extra trouble and expense to have everything as nice, if not a little *nicer* than her neighbors. I tell you it's a downright trouble and vexation to prepare tea for company, and I become so tired after it, that I cannot half enjoy their visit."

"Well, but wife, what is the necessity for us doing as other people do? Surely Mr. and Mrs. B. will not expect us to set a grand, magnificent table, simply because we invite them in to take tea and spend a few hours in conversation with us. The intention is not to fill their stomachs, and render them uncomfortable with rich and indigestible food, but rather to have a quiet sociable visit with them."

"True, but wherever they have been invited to tea, there have they witnessed the evident effort of the housekeeper to have everything better than usual. The children have been s uced up—the parlor is in perfect order—the bed in the front chamber, where it is expected they will lay off their wraps, and arrange their *toilet*, must have a clean white spread and pillow-cases. In short, the entire house must undergo re-arrangement, to say nothing of the extra outlay of labor in preparing the tea table, and all this must be done before they can entertain their friends at tea."

"Wife."

"Well."

"Don't let's ask anybody to tea."

"Why?"

"O, gracious, I had no idea that all you have just mentioned had to be done. I supposed that a clean cloth, two extra plates, some brown bread, a little good butter, a nice cup of tea, some light biscuit, and a few slices of cold ham, were all that is necessary to ensure one a real nice, palatable supper. But if you're going into a general house cleaning at this season of the year, and intend getting up a banquet that would astonish a conclave of Knights Templar, why that's a different matter—I withdraw the proposition."

"Please spare your sarcasm, sir, for you know as well as I do, that just so much preparation is necessary, and that I'm placed in a perfect flurry of excitement from the minute company is mentioned until the affair is over with. It's a very easy and pleasnt thing for you men to invite company to the house, but I doubt whether you ever once think of the real hard labor you subject your wives to, in so doing."

"Say, wife."

"Well, what!"

"Let me tell you what *I* think about it."

"O, yes; I'll warrant you're prepared with some wonderful philosophical argument, that will do very well to listen to, but will not bear the test of experiment. I tell you tea-parties are a bother and an abomination, and you can't make anything else out of them."

"O, yes we can, wife!"

"How, I should like to know? Please invent a plan, and you shall be blessed among women."

"Well, I'll tell you. *Always* have your house clean and neat—see that it is swept regularly once every week, from garret to cellar. *Always* have a clean cloth upon the table, and an extra plate *always* at my right for any friend that may chance to accompany me home to dinner or tea. Then, you see, you will never know when your company is coming, and still will be *always* ready to receive them, for I contend that my table is *always* good enough for my friends. So, look out wife, when you least expect it, then will your company come, and I doubt not at such times you will be able to sit down and have a pleasant meal and chat, without making yourself sick *trying* to do the very same thing on notification."

[NOTE.]

Wife thinks she'll try the experiment, and in the July number of the BISTOURY we may tell our readers how she succeeded with her tea-parties under the new order of things.

